# Pulmonary Vein Index Is Associated With Early Prognosis of Surgical Treatment for Tetralogy of Fallot

**DOI:** 10.3389/fped.2021.705553

**Published:** 2021-07-12

**Authors:** Haoyong Yuan, Tao Qian, Ting Huang, Hui Yang, Can Huang, Ting Lu, Zhongshi Wu

**Affiliations:** ^1^Department of Cardiovascular Surgery, The Second Xiangya Hospital of Central South University, Changsha, China; ^2^Engineering Laboratory of Hunan Province for Cardiovascular Biomaterials, Changsha, China; ^3^Department of Radiology, The Second Xiangya Hospital of Central South University, Changsha, China

**Keywords:** tetralogy of Fallot, pulmonary vein index, McGoon ratio, early outcomes, congenital heart disease

## Abstract

**Objectives:** To evaluate the predictive value of the pulmonary vein index (PVI) in the early prognosis of patients who received total tetralogy of Fallot (TOF) repair.

**Methods:** We retrospectively reviewed 286 patients who underwent TOF repair in our institution between July 2013 and May 2020. The PVI, McGoon ratio, and Nakata index were measured and calculated. Logistic regression, linear stepwise regression, receiver operating characteristic (ROC) curve analysis, and Cox proportional hazards modeling were performed to evaluate the predictive value of PVI in the early prognosis after TOF repair surgery.

**Results:** The median age and body weight were 1.23 (0.22–15.02) years and 9.00 (3.00–44.00) kg, respectively. There were five early deaths. The areas under the ROC curve for death were 0.89, 0.79, and 0.88 for the McGoon ratio, Nakata index, and PVI, respectively. A lower PVI better predicted prolonged postoperative hospital stay, cardiac intensive care unit stay, and ventilator time (Hazard Ratio, HR [95% Confidence intervals, CI]: 1.003 [1.002–1.004], *p* < 0.001; 1.002 [1.001–1.004], *p* < 0.001; 1.002 [1.001–1.003], *p* < 0.001, respectively) and was a significant risk factor for high 24 h max Vasoactive inotropic score (Crude Odds Ratio [OR] [95% CI]: −0.015 [−0.022, −0.007], *p* < 0.001), serous effusion (Crude OR [95% CI]: 0.996 [0.992–0.999], *p* = 0.020), delayed sternal closure (Crude OR [95% CI]: 0.983 [0.971–0.996], *p* = 0.010), and the need for peritoneal dialysis (Crude OR [95% CI]: 0.988 [0.980–0.996], *p* = 0.005). The area under the ROC curve of PVI for delayed postoperative recovery was 0.722 (*p* < 0.001), and the estimated cutoff point was 300.3 mm^2^/m^2^.

**Conclusion:** PVI is a good predictor of early prognosis for surgical treatment of TOF patients.

## Introduction

Treatments for tetralogy of Fallot (TOF) have achieved significant success over the past decades (1, 2). The perioperative mortality has decreased to as low as ~1.5% along with better long-term outcomes (1), which is attributed to the evolution of surgical techniques and improvements in cardiopulmonary bypass and perioperative management (3, 4). However, TOF patients are remarkably clinically heterogeneous, which may lead to misgivings when making treatment decisions and predicting the prognosis early. Although, the postoperative course is usually uneventful in most patients, a subset of patients experiences a prolonged postoperative recovery in the absence of any residual (correctable) surgical lesions (5, 6).

The decrease in pulmonary blood flow (PBF) is the main hemodynamic change in TOF. Indicators currently used for PBF evaluation are based on the size of the pulmonary arteries (PAs), such as the McGoon ratio and Nakata index. However, such indicators have some limitations. The diameter of the PAs measured on computed tomography angiography (CTA), magnetic resonance (MR) imaging, or ultrasound are not accurate since PAs are not typically round. In addition, the presence of collateral vessels, poststenotic dilation and malformation may lead to misjudgments of PBF and further influence the estimation of the illness condition and prognosis and the determination of treatment decisions.

It has been demonstrated that the size of the pulmonary veins (PVs) is a more accurate indicator of PBF than the size of the PAs size, especially in the TOF cohort (7, 8). Considering that PBF is negatively associated with the severity of TOF in patients, a novel indicator based on PV morphology may have the potential to provide a more precise prediction. However, the prognostic value of the pulmonary vein index (PVI, defined as the total PV area divided by the body surface area) in TOF patients has not yet been studied.

In this study, we retrospectively reviewed TOF patients who underwent complete repair in our institution and aimed to evaluate the correlations between PVI and early outcomes after TOF repair and compare them with those of the McGoon ratio and Nakata index.

## Patients and Methods

### Patients

This study was approved by the Institutional Ethics Committee of the Second Xiangya Hospital, Central South University. Written informed consent was obtained from the parents or guardians before surgery to allow the use of patient data. A total of 332 consecutive TOF patients underwent repair surgery from July 2013 and May 2020 in our institution. Patients with Down's syndrome (*n* = 2), pulmonary atresia (*n* = 32), absence of pulmonary valves (*n* = 2), complete atrioventricular septal defects (*n* = 2), total anomalous pulmonary venous connections (*n* = 3), and no available CTA data (*n* = 5) were excluded. Finally, 286 patients were enrolled in this study. The preoperative data are shown in Table 1.

**Table 1:** Information of patients.

**Basic Characteristics**	
Age, years	1.23 (0.22–15.00)
Weight, kg	9.00 (3.00–44.00)
Famale/male, *n*	112/174
Preoperative oxygen saturation	86.76 ± 10.43
Previous surgical procedures, *n*	
Modified Blalock-Taussig shunt	2
PV balloon and stent implantation	1
McGoon ratio	1.84 ± 0.34
Nakata index, mm^2^/m^2^	232.32 ± 87.08
PVI, mm^2^/m^2^	358.95 ± 115.65
**Operative data**
CPB time, min	95.68 ± 39.97
ACC time, min	61.04 ± 21.20
Surgical strategy, *n* (%)	
Without TAP	130 (45.4)
With TAP	156 (54.6)
Associated procedures	
ASD repair	72
PDA ligation	26
Muscular VSD occlusion	1
MAPCAs	60
Occlusion	17
Ligation	3

*The basic characters, operative, and postoperative information of the patients. PV, Pulmonary valve; PVI, Pulmonary vein index; CPB, Cardiopulmonary bypass; ACC, Aortic cross-clamping; TAP, Trans-annular patch; ASD, Atrial septal defect; PDA, Patent ductus arteriosus; VSD, Ventricular septal defect; MAPCAs, Major aortopulmonary collateral arteries; RVOT, Right ventricular outflow tract; RVP/LVP, Postoperative right and left ventricle pressure ratio; CICU, Cardiac intensive care unit; AVB, Atrioventricular block*.

### CTA Data Collection and Analysis

All CTA data were obtained by a third-generation dual-source CT scanner (SOMATOM Definition Force, Siemens Healthcare, Forchheim, Germany). Lead aprons were used to protect the thyroid and gonads for all patients. Prospective electrocardiographic gating was performed to eliminate cardiac motion artifacts. After scanning, the data were reconstructed at 0.5 mm intervals.

The CTA data were analyzed using Mimics Research software version 21.0 (Materialise, Leuven, Belgium) by two independent radiologists who were blinded to each other's results. The reliability of the measurements from the two researchers was evaluated with two-way interclass correlation mixed with absolute agreement and is expressed as intraclass correlation coefficients (ICCs) and 95% confidence intervals (CIs). An ICC >0.7 was considered valid (Supplementary Table 1). The diameters of the PAs and descending aorta were measured on the axial plane (Supplementary Figures 1A–C). The cross-sectional areas of the PAs and PVs were semiautomatically measured on the 3-dimensional view reconstructed by the software (Supplementary Figures 1D,E). The area of the PAs was measured at the proximal point of the first branch. The area of the PVs (LPV-A, RPV-A) was measured 3 mm beyond the pulmonary venous ostium. The McGoon ratio and Nakata index were calculated using classical methods (9, 10). PVI was calculated by the following formula:

$$\begin{array}{l} \text{PVI}=\;\frac{\text{LPV}-\text{A}+\text{RPV}-\text{A}}{\text{BSA}}\left(\text{m}{\text{m}}^{2}/{\text{m}}^{2}\right) \end{array}$$

### Surgical Techniques

The operation was performed with full-flow cardiopulmonary bypass and moderate hypothermia with repeated crystalloid cardioplegia under general anesthesia. We performed the repair without a trans-annular patch (TAP) on 130 patients, among whom 88 underwent trans-atrial repair and 42 underwent repair with an additional right ventricular outflow tract (RVOT) incision. The other 156 patients, who had a pulmonary artery annulus Z score <-4 and right ventricular infundibulum dysplasia, underwent RVOT augmentation with a trans-annular bovine pericardium patch (Supplementary Figure 2). Transesophageal echocardiography was performed to evaluate the correction of intracardiac deformities before cardiopulmonary bypass was removed. A right and left ventricular pressure ratio (RVP/LVP) <0.7 was considered acceptable (Table 2).

**Table 2:** Postoperative information.

RVOT peak gradient (mmHg)	27.32 ± 14.31
RVP/LVP	0.54 ± 0.13
Postoperative hospital stay (days)	12.00 (4.00–97.00)
Mechanical ventilation time (hours)	19.71 (3.12–333.49)
CICU stay (hours)	66.93 (5.98–477.07)
24 h max vasoactive inotropic score	12.35 (4.00–56.00)
Major complications, *n* (%)	
Serous effusion	58 (20.28)
III^°^AVB	2 (0.70)
Bleeding requiring resternotomy	3 (1.05)
Delayed sternal closure	18 (6.29)
Renal failure requiring temporary dialysis	15 (5.24)
Nervous system complications	5 (1.75)

*The postoperative information of the patients. RVOT, Right ventricular outflow tract; RVP/LVP, Postoperative right and left ventricle pressure ratio; CICU, Cardiac intensive care unit; AVB, Atrioventricular block*.

All patients were admitted to the cardiac intensive care unit (CICU) after surgery and supported by mechanical ventilation and vasoactive agents according to our standard protocol. Early death was defined as occurring within 30 days postoperatively.

### Statistical Analysis

Data were tested for a normal distribution with the Shapiro-Wilk test. Continuous variables with a normal distribution are presented as means ± standard deviations, and variables with a skewed distribution are presented as medians with ranges. Categorical variables are presented as frequencies and percentages. Data were analyzed using SPSS 25.0 (SPSS Inc., Chicago, Ill) and Prism 8.0 (GraphPad Software, San Diego). Correlations between variables were analyzed using a linear correlation test. The predictive value for early death and delayed recovery was analyzed using receiver operating characteristic (ROC) curves. Delayed recovery was defined a death or the third quartile of postoperative hospital stay (PHS), CICU stay, and ventilator time. Youden's index was calculated to estimate the cutoff point. A Cox proportional hazards model was used to analyze risk factors for PHS, CICU stay, and ventilator time. Cox proportional hazards are semiparametric because they make specific assumptions about the probability distribution of event times. Linear regression and logistic regression were performed for risk factors for high vasoactive inotropic score (VIS) and major postoperative complications, respectively. Variables examined in univariable analysis were enrolled in the multivariate model if *p* < 0.20. The McGoon ratio and Nakata index were highly correlated, and most of the treatment decisions were made based on the McGoon ratio; therefore, the McGoon ratio was retained in the multivariate model. Covariates for the multivariate analysis were also selected a priori based on clinical plausibility and data availability and retained for clinical or statistical significance. *p* < 0.05 was considered statistically significant.

## Results

### PA and PV Morphology

The CTA data are shown in Table 1. Five patients had stenosis at the orifice of the left/right PA and apparent poststenotic dilation (Supplementary Figures 3D,E). Augmentation of the stenotic PA branch was performed from the proximal to distal end. There were two main kinds of PV anatomical variants, but no pathological abnormities were found. In 17 patients, a single left pulmonary vein was revealed (Supplementary Figure 3F). A separate right middle lobe vein appeared in 21 patients (Supplementary Figure 3G).

**Figure 1:**
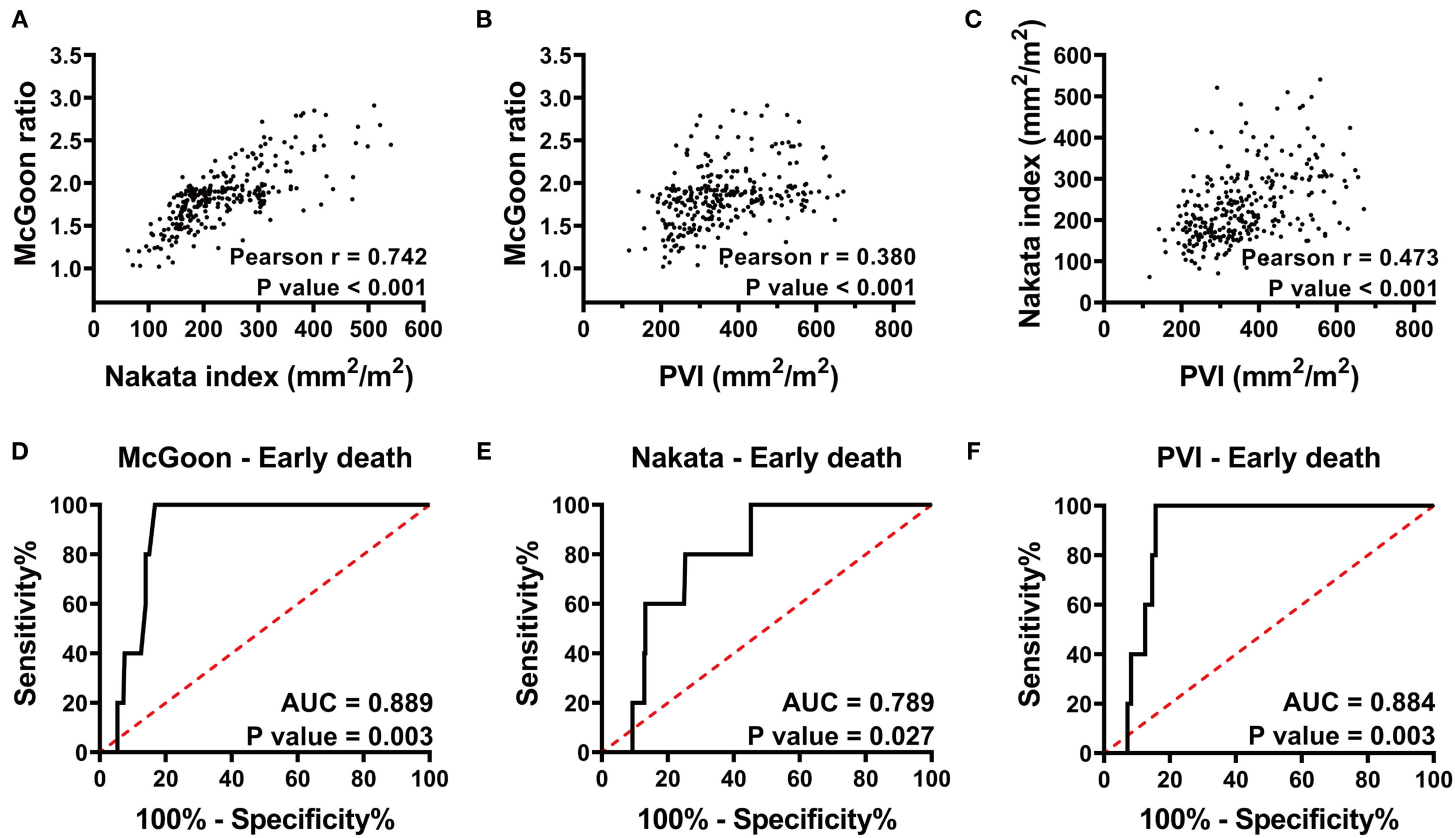
**(A–C)** Pearson correlation analysis of the McGoon ratio, Nakata index, and PVI indicate that the McGoon ratio has a strong correlation with the Nakata index, whereas, the correlation between the PVI and the two PA parameters was weak. **(D–F)** Receiver operating characteristic curve analysis indicates the predictive value of the McGoon ratio, Nakata index, and PVI for early death. PVI has a predictive value similar to that of the McGoon ratio and much better than that of the Nakata index. r, Pearson correlation coefficient; AUC, area under the ROC curve; PVI, pulmonary vein index. *p* < 0.05 indicates statistical significance.

There was a strong correlation between the McGoon ratio and Nakata index. However, the correlations between PVI and the two PA parameters were weak (Figures 1A–C; Pearson's r: 0.742, 0.380, and 0.473, respectively, *p* < 0.001).

### PVI and Early Death

There were 5 early deaths, resulting in a mortality rate of 1.7% (Table 3). One patient, with an RVP/LVP ratio of 0.9 after augmentation of the RVOT, died of right heart failure within 48 h. One patient died of uncertain sudden death on the first postoperative day. One patient died of acute left heart failure, and one died of multiple organ dysfunction secondary to low cardiac output syndrome. The last patient died of severe infection and respiratory failure. The areas under the ROC curves (AUCs) of the McGoon ratio, Nakata index, and PVI were 0.89 (*p* = 0.003), 0.79 (*p* = 0.027), and 0.88 (*p* = 0.003), respectively (Figures 1D–F). The results indicated comparable predictive value among the PVI, McGoon ratio, and Nakata index for early death.

**Table 3:** Summarize of early mortalities.

**No**.	**Age (years)**	**Weight (kg)**	**McGoon**	**Nakata (mm^**2**^/m^**2**^)**	**PVI (mm^**2**^/m^**2**^)**	**Surgery**	**RVP/LVP**	**Cause of death**
1	1.19	8.00	1.49	182.19	213.21	TAP	0.70	LCOS/MODS
2	1.40	7.00	1.37	175.72	244.11	TA	0.60	Sudden death
3	2.30	9.00	1.48	211.50	233.62	TA	0.70	ALHF
4	1.08	7.20	1.27	254.65	239.01	TAP	0.90	Right heart failure
5	0.27	5.40	1.52	202.40	220.58	TAP	0.55	Infection/RF

*PVI, Pulmonary vein index; RVP/LVP, Postoperative right and left ventricle pressure ratio; TAP, Trans-annular patch; TA, Trans-atrial; LCOS, Low cardiac output syndrome; MODS, Multiple organ dysfunction syndrome; ALHF, Acute left heart failure; RF, Respiratory failure*.

### PVI and Postoperative Recovery

Among the 281 survivors, the PHS, CICU stay, mechanical ventilator time, and 24-h maximum vasoactive inotropic score (VIS) are shown in Table 2. The Cox proportional hazards model showed that lower PVI was a significant risk factor for prolonged PHS, CICU stay, and ventilator support (Table 4 and Supplementary Table 2). For each 100 mm^2^/m^2^ increase in PVI, the rates of discharge from the hospital, discharge from the CICU, and extubation increased by 30, 20, and 20%, respectively. However, the McGoon ratio and Nakata index were not associated with these three postoperative parameters. Lower PVI, lower body weight, longer CPB time, preoperative SPO_2_, and postoperative RVP/LVP were associated with a higher VIS. A low PVI was the most significant preoperative risk factor for high VIS (Table 5 and Supplementary Table 3).

**Table 4:** Multivariate analysis of risk factors for postoperative parameters.

**Variables**	**PHS**		**CICU Stay**		**Ventilator time**	
	**HR (95% CI)**	***P*-value**	**HR(95% CI)**	***P*-value**	**HR(95% CI)**	***P*-value**
**Weight (kg)**	1.030 (1.011–1.050)	0.002	1.110 (1.088–1.133)	<0.001	1.080 (1.059–1.101)	<0.001
**PVI**	1.003 (1.002–1.004)	<0.001	1.002 (1.001–1.004)	<0.001	1.002 (1.001–1.003)	0.001
**CPB time**	0.992 (0.88–0.996)	<0.001	0.985 (0.978–0.992)	0.001	0.986 (0.982–0.991)	<0.001
**MAPCAs treatment**						
**No**	Reference					
**Yes**	1.464 (0.899–2.381)	0.125	0.299 (0.15–0.508)	<0.001	0.326 (0.12–0.555)	<0.001
**Postoperative RVP/LVP**	0.408 (0.146–1.141)	0.088	0.181 (0.065–0.506)	0.001	0.142 (0.053–0.376)	<0.001

*Results of multivariate Cox proportional hazards analysis for PHS, CICU Stay, and Ventilator time were reported by HR (Hazard ratios) and 95% CI (Confidence intervals). PHS, Postoperative hospital stay; CICU, Cardiac intensive care unit; PVI, Pulmonary vein index; CPB, Cadiaopulmonary bypass; MAPCAs, Major aortopulmonary collateral arteries; RVP/LVP, Postoperative right and left ventricle pressure ratio. p < 0.05 indicates statistical significant variables in multivariate analysis*.

**Table 5:** Linear stepwise regression analysis of risk factors for VIS.

**Variables**	**Crude OR (95% CI)**	**Adjusted OR**	***P*-value**
Weight (kg)	–0.200 (–0.337, –0.062)	–0.141	0.005
PVI	–0.015 (–0.022, –0.007)	–0.222	<0.001
CPB time	0.074 (0.050, 0.098)	0.319	<0.001
Preoperative SPO_2_	–0.088 (–0.172, –0.003)	–0.120	0.043
Postoperative RVP/LVP	8.282 (2.069, 14.495)	0.138	0.009

*Results of Linear stepwise regression analysis for VIS were reported by Crude OR (Odds ratios), 95% CI (Confidence intervals), and Adjusted OR. Variables that significantly correlated with VIS were selected for stepwise regression (Supplementary Table 2). PVI, Pulmonary vein index; CPB, Cadiaopulmonary bypass; MAPCAs, Major aortopulmonary collateral arteries; RVP/LVP, Postoperative right and left ventricle pressure ratio*.

Fifty-eight patients suffered serous effusion in this cohort, including 1 chylothorax, 2 pericardial effusion, and 55 pleural effusions. Complete atrioventricular block occurred in 2 patients, and their temporary pacemakers were removed during the CICU stay. Eighteen patients suffered delayed sternal closure. Peritoneal dialysis was required in 15 patients. Logistic regression was performed for the three major postoperative complications: serous effusion, delayed sternal closure, and the need for peritoneal dialysis. PVI served as the only preoperative risk factor for serous effusion. For each 100 mm^2^/m^2^ increase in PVI, the rate of serous effusion decreased by 40% (Table 6 and Supplementary Table 4). PVI also showed a significant association with delayed sternal closure and the need for peritoneal dialysis (Table 6 and Supplementary Table 4).

**Table 6:** Multivariate analysis of Risk factors for major postoperative complications.

**End points**	**Variables**	**Crude OR (95% CI)**	***P*-value**
Serous effusion	PVI	0.996 (0.992–0.999)	0.020
	CPB time	1.011 (1.002–1.020)	0.018
Delayed sternal closure	Weight	0.48 (0.309–0.746)	0.001
	PVI	0.983 (0.971–0.996)	0.010
	Clamping	1.035 (1.007–1.064)	0.013
	Postoperative RVP/LVP	992.756 (2.679–317837.463)	0.022
Need for peritoneal dialysis	Weight	0.696 (0.510–0.950)	0.023
	PVI	0.988 (0.980–0.996)	0.005

*Results of multivariate logistic regression analysis for major postoperative complications were reported as Crude OR (Odds ratios) and 95% CI (Confidence intervals). Univariate analysis was shown in Supplementary Table 3. PVI, Pulmonary vein index; CPB, Cadiaopulmonary bypass; RVP/LVP, Postoperative right and left ventricle pressure ratio*.

**Figure 2:**
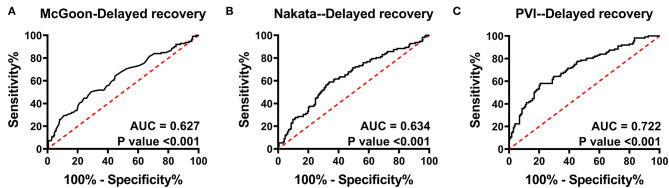
Receiver operating characteristic curve analysis for delayed postoperative recovery. **(A–C)** indicate the predictive value of the McGoon ratio, Nakata index, and PVI for delayed recovery, respectively. The AUC of PVI was the largest. AUC, area under the ROC curve; PVI, pulmonary vein index. *p* < 0.05 indicates statistical significance.

In total, 112 patients suffered delayed postoperative recovery. The ROC curves of delayed postoperative recovery are shown in Figure 2. The AUCs of the McGoon ratio, Nakata index, and PVI were 0.627, 0.634, and 0.722, respectively (*p* < 0.001). PVI showed the best predictive value for delayed recovery. The estimated cutoff point of PVI was 300.3 mm^2^/m^2^.

## Discussion

**Figure 3:**
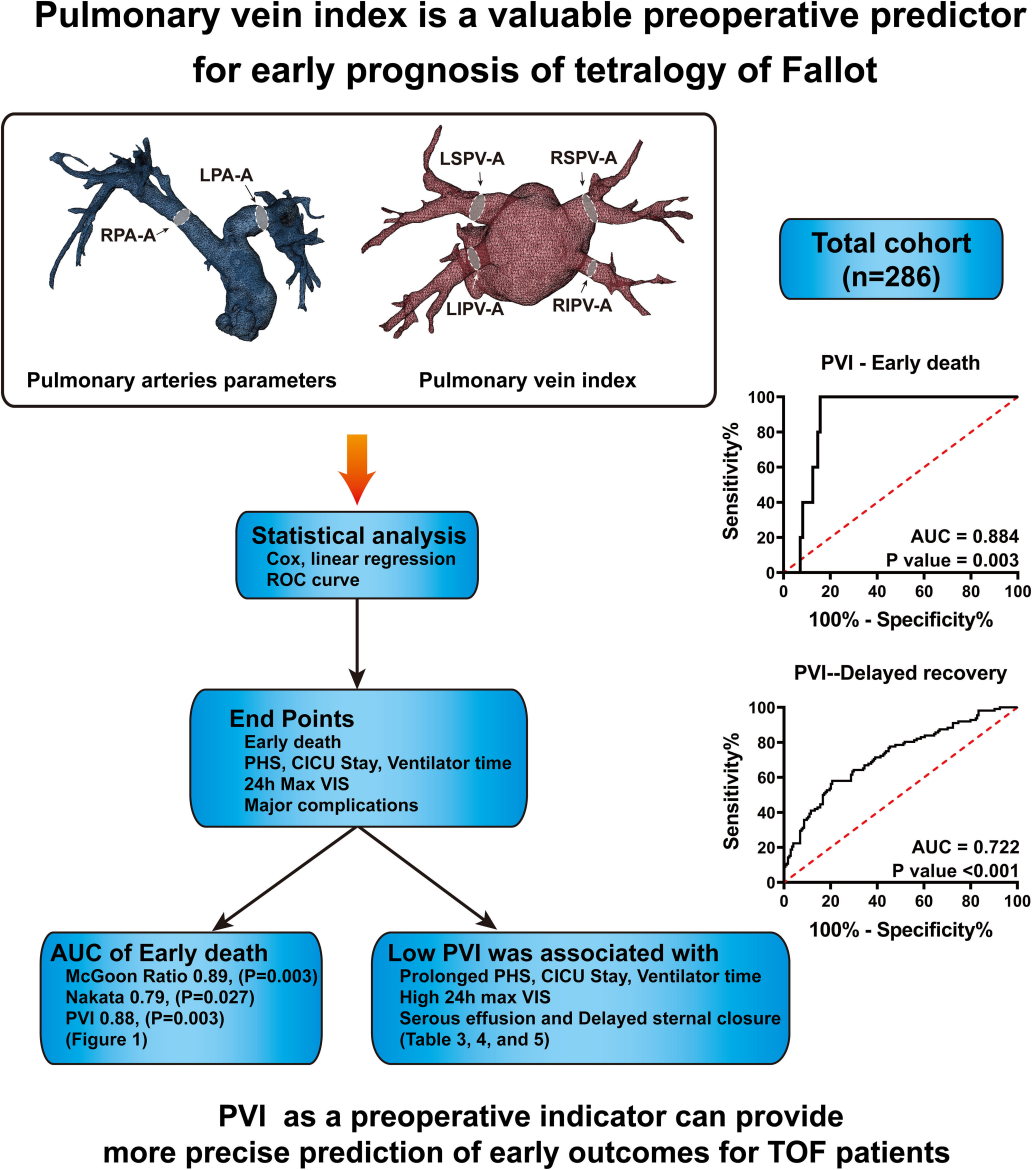
In total, 286 patients' CTA data were collected and analyzed. The sizes of pulmonary arteries and veins were measured, and the McGoon ratio, Nakata index and pulmonary vein index were calculated. ROC curve analysis demonstrates that PVI has a similar predictive value for early death as the McGoon ratio and Nakata index. PVI has better predictive value for delayed postoperative recovery.

This study aimed to determine the predictive value of PVI for early outcomes among surgical treatment of TOF patients. Our results support the conclusion that low PVI is a significant risk factor for early death and prolonged postoperative recovery (Figure 3).

### Why CTA Data Were Chosen

CTA is recommended since it can reveal the morphology of extracardiac vasculature, including the coronary vessels, PAs, aorta, and pulmonary or systemic veins (11). In our institution, a series of protective procedures are performed for every child undergoing CTA scans. The main reasons for not choosing MR imaging are the high patient heart rates, the much longer scanning times, and the need for complete sedation or anesthesia. In addition, both CTA and MR imaging are routinely used to measure PV sizes, with measurements being comparable between the two methods (11, 12). Nevertheless, CTA offers a speed advantage and has greater spatial resolution (12).

The development of high-resolution CTA has provided us with a more convenient method to depict the PV anatomy. Three-dimensional reconstruction technology further visualizes the structure of the PVs. In most studies, the areas of the PVs were measured by manually drawing the region of interest from a cross-sectional view (8, 12). This may lead to systemic errors and a high requirement of manual work. In our study, the measurements of the PVs were performed on a 3D view with an automatically calculated centerline that indicated the direction of blood flow. A cross-section perpendicular to the centerline was selected automatically, which might be more accurate and accessible. However, here, we advocate the concept of using the PV index to achieve a more precise prediction. For measurement methods, CTA, MRI, and echocardiography data would be good choices so long as the data are accurate.

### Treatment Management and Early Outcomes of TOF Patients

Treatments for TOF have been increasingly performed in recent decades, and early surgical repair can be achieved with low mortality and excellent long-term results in many institutions (6, 13, 14). Herein, we achieved an early mortality as low as 1.7%, which is analogous with the findings of other studies (15, 16). Low mortality is not a hot topic of current clinical research, and surgeons tend to focus more on providing meticulous management before and after the operation. Our results showed that a lower PVI has the potential to be a precise indicator for early outcomes, which is beneficial for starting intensive management ahead of time. Identifying risk factors for perioperative outcomes after complete repair of TOF might identify opportunities to modify early and late outcomes (17).

In this cohort, 130 (45.4%) patients underwent repair without TAP augmentation, which is similar to or even better than the results reported by other centers (18–20). However, it is thought that the placement of the TAP often results in relatively delayed postoperative recovery after surgical repair (17). We believe that our results are representative, considering the similar proportions of different surgical strategies with those of other studies.

In TOF, the postoperative hospital course can vary in terms of length of mechanical ventilation, postoperative complications, and PHS (21, 22). PHS is one of the most important outcome measures for congenital cardiovascular surgery (17, 23). Herein, we reported a mean PHS of 12 (4–97) days, which is longer than the seven (interquartile range 4–12) days and eight (interquartile range, 6–10) days reported by Mercer-Rosa et al. (17) and Lodin et al. (21), respectively. The main reason is that they defined discharge as release either to home or to a chronic care facility. However, chronic care facilities are rare in developing countries such as China. Fifty-eight patients suffered serous effusion, which was the most common complication after surgical repair. The incidence (20.28%) is higher than the 13% reported by Mouws et al. (20). This may be associated with the differences between the two study populations.

### Predictive Value of PVI vs. PA Parameters

PBF reflects the functionality of the entire pulmonary vasculature, which significantly affects the prognosis of patients with cyanotic congenital heart diseases. PA and PV parameters are the most commonly used parameters for tracing PBF (7). PA parameters have been widely used as indications for the surgical repair of TOF. However, to our knowledge, this is the first study to focus on the predictive value of PVI for the early outcomes of TOF repair.

The PV area may provide important information about the difference in blood flow and vascular resistance between each lung in patients with congenital heart disease (8). In this study, weak correlations between PVI and PA parameters were demonstrated by linear correlation test. That might be due to the limitations of PA parameters. In patients with many collaterals from the systemic circulation to the PAs or patients with PA deformities, the diameter of the PAs does not ideally reflect PBF (8). Instead, the PVs are the unique exits of PBF, even considering collaterals, and PV deformities have rarely been reported in TOF cohorts. In addition, pulmonary venous flow is more constant than pulmonary arterial flow (24). The size of the PVs may be directly related to PBF.

In this study, PVI showed similar predictive value for early death with the McGoon ratio and Nakata index, according to the area under the ROC curve (Figures 1D–F). PA size is a crucial prognostic factor in managing TOF because there is a risk of right ventricular failure if the outflow resistance remains too high after separating the pulmonary and systemic circulations (25). However, Groh et al. (26) revealed that there is a weak correlation with considerable scatter between PA size and postrepair RVP/LVP. Therefore, combining PV indicators might be an excellent choice for improving the accuracy of the evaluation. However, the limited number of cases of death might influence the results, and further study is needed to understand the basis for this.

**Figure 4:**
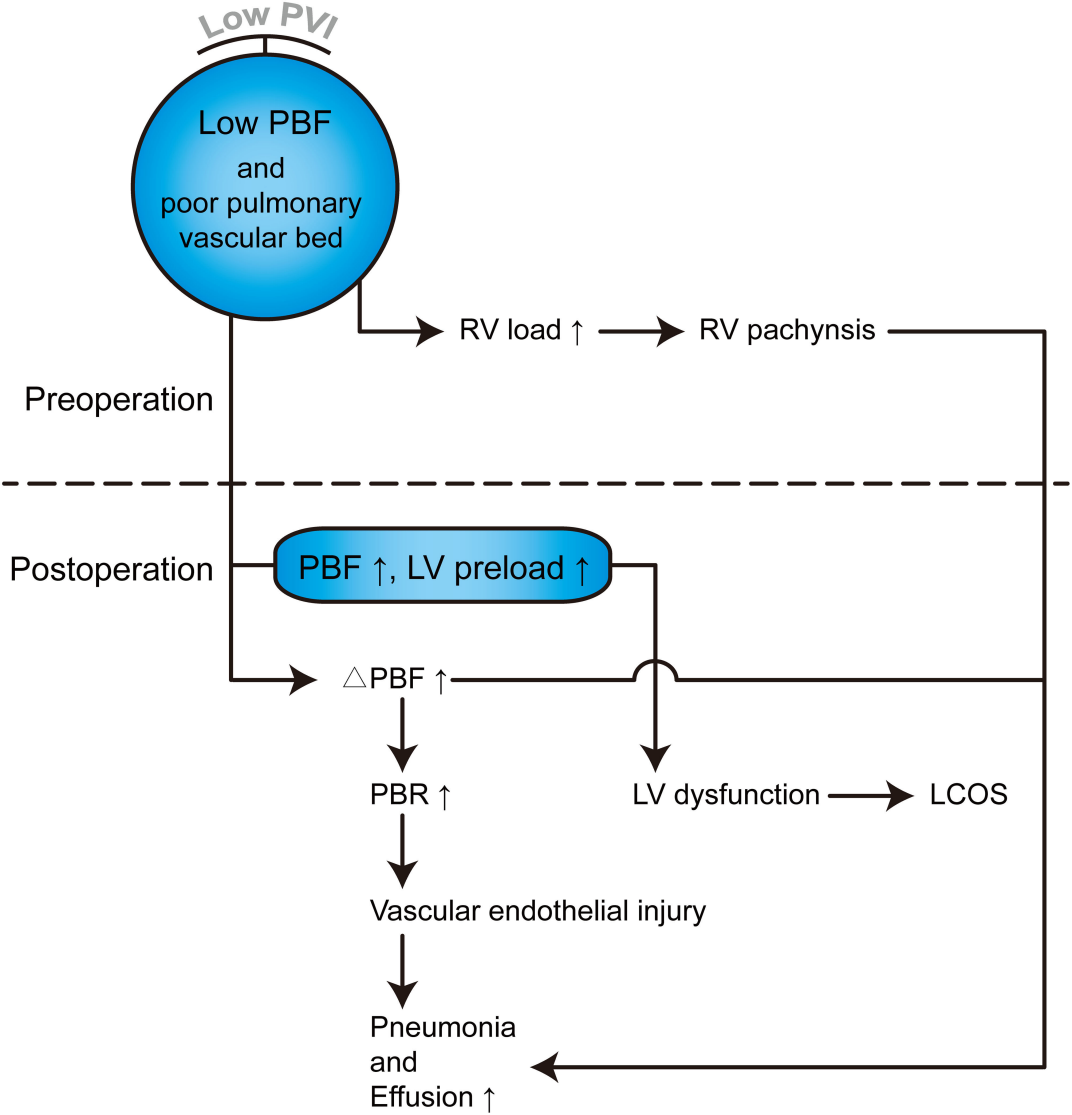
Hemodynamic and pathophysiological changes in TOF patients with lower PVI. PVI can reflect the total PBF and pulmonary vascular bed, which can further reflect left and right heart pathophysiological changes. PBF, Pulmonary blood flow; RV, Right ventricle; LV, Left ventricle; ΔPBF, change in PBF postoperatively; PBR, Pulmonary blood flow rate; LCOS, Low cardiac output syndrome.

PHS is one of the most important parameters for the assessment of operative outcomes when postoperative mortality is low (17). Even one additional day in the hospital is associated with significant morbidity and excess costs (27). A long PHS is not only a metric of early outcome but is also associated with long-term neurodevelopmental outcomes in children with congenital heart defects (28, 29). Our results indicated that PVI served as the best predictor of PHS, outperforming the McGoon ratio and Nakata index (Table 4). A lower PVI was also associated with longer CICU stay, longer mechanical ventilator support, and higher VIS. However, whether PA size is associated with these postoperative parameters remains controversial, and thus, our ability to identify patients at risk of prolonged PHS based on preoperative PA size is limited by conflicting evidence in the literature (17, 26). The possible reason is that the PA size was within normal limits, although, both the left and right PA were small (17). However, as the PVs are the only exit of PBF, the PVI reflects the total PBF, which may be highly associated with postoperative recovery and morbidity. Studies on major postoperative complications have demonstrated that a lower PVI is associated with a high risk of serous effusion, delayed sternal closure, and the need for peritoneal dialysis (Table 6 and Supplementary Table 4), which may undoubtedly increase the lengths of PHS, CICU stay, and ventilator support. A lower PVI indicates lower PBF and relative pulmonary vascular bed dysplasia, which may lead to a higher right ventricle preload and afterload and a relatively lower return blood flow from the PVs to the left heart. After repair, the increased PBF results in a tremendous enhancement of left ventricle preload, which may have a more significant influence for low PVI patients, manifesting as left or right heart dysfunction. Additionally, the rebalance of blood flow causes a much more substantial change in PBF for low PVI patients and further results in a much higher PBF velocity in the lung. This may lead to vascular endothelial injury, and pneumonia and serous effusion may occur. Furthermore, the higher right ventricle load results in more severe right ventricle pachynsis, which may lead to a higher right atrium/superior vena cava pressure and play a critical role in postoperative complications (Figure 4). In our study, body weight at surgery was associated with PHS, CICU stay, and ventilator time (Table 4), which is similar to the results reported by some groups (30, 31) but different from those reported by Mercer-Rosa et al. (17). This may be because we had a more significant variation in age and body weight in our cohort, which is a common situation in most developing countries. However, the PVI was already adjusted by BSA, which may be more useful for all age ranges. A lower PVI may indicate much more difficult postoperative recovery, which should be taken into account well by physicians and surgeons to avoid adverse events.

The AUCs of the McGoon ratio, Nakata index, and PVI for delayed recovery demonstrated that the PVI has the best predictive value (Figure 2). Here, we defined delayed recovery by the third quartile of PHS, CICU stay, and ventilator time, as Mercer-Rosa et al. (17) did in their recent study, and estimated a cutoff point of 300.3 mm^2^/m^2^ of PVI. This result indicates that a patient with a PVI <300 mm^2^/m^2^ is more likely to suffer delayed postoperative recovery.

## Limitations

This study has a single-center retrospective design with a limited sample size. Although, a total of 286 patients were enrolled in this study, there were only 5 deaths. Therefore, we cannot give an exact cutoff point for PVI in predicting early death. The evidence quoted for the validity of using PVI was limited. Furthermore, this study only focused on early outcomes of TOF repair, and the predictive value of PVI for long-term outcomes or surgical strategy decisions was not determined. Our future work will focus more on these aspects.

## Conclusion

This study demonstrated that PVI is a risk factor for early death after TOF repair and a good predictor of early prognosis for TOF patients. These findings may provide more accurate information for aiding in improving postoperative management.

## Data Availability Statement

The original contributions presented in the study are included in the article/Supplementary Material, further inquiries can be directed to the corresponding author/s.

## Author Contributions

ZW and CH provided the idea and concept of this study. HY contributed to patient data collection, CTA data analysis, statistic analysis, and writing. TQ contributed to CTA data analysis and statistic analysis. TH and HY was in charge of CTA data collection and analysis. TL contributed to manuscripts editing and revising. All authors contributed to the article and approved the submitted version.

## Conflict of Interest

The authors declare that the research was conducted in the absence of any commercial or financial relationships that could be construed as a potential conflict of interest.

## Supplementary Material

Supplementary Figure 1 Measurement of PA diameter, aorta diameter, PA area, and PV area. **(A)** Diameters of the left and right pulmonary arteries (LPA-D, RPA-D), measured in the axial plane. **(B)** Diameter of the descending aorta (D-AO) at the diaphragmatic level in the axial plane. **(C)** Depicts the measurement position for the descending aorta on the sagittal plane. **(D)** Areas of the left and right pulmonary arteries (LPA-A, RPA-A) on a 3D reconstructed view. **(E)** Areas of the left and right superior PV (LSPV-A, RSPV-A) and left and right inferior PV (LIPV-A, RIPV-A) on the 3D reconstructed view.

Supplementary Figure 2 Schematic diagram showing our surgical repair strategy for TOF. RVOT, right ventricle outflow tract; VSD, ventricular septal defect; PV, pulmonary valve; PVA, pulmonary valve annular; PA, pulmonary artery; CPB, cardiopulmonary bypass.

Supplementary Figure 3 The first branch of the left pulmonary artery commonly surrounds the back of the left superior pulmonary vein and moves toward the outside **(A)**. The right superior and inferior pulmonary arteries originate inside the pericardia and moved upward and downward, respectively **(B,C)** which may be difficult to distinguish in the axial view. **(D,E)** indicate apparent stenosis and stenotic dilation. **(F)** indicates a single left pulmonary vein. **(G)** indicates a separate right middle lobe vein.

Supplementary Table 1 Reliability analysis of CTA measurements with same methods between two independent radiologists.

Supplementary Table 2 Univariate analysis of Risk factors for postoperative parameters.

Supplementary Table 3 Linear stepwise regression analysis of risk factors for VIS.

Supplementary Table 4 Univariate logistic regression analysis of Risk factors for major postoperative complications.
