# Supplementary figures and images for: Pulmonary Vein Index Is Associated With Early Prognosis of Surgical Treatment for Tetralogy of Fallot
Source: Front Pediatr. 2021 Jul 12;9:705553. doi: 10.3389/fped.2021.705553 (PMC8311495; doi:10.3389/fped.2021.705553)

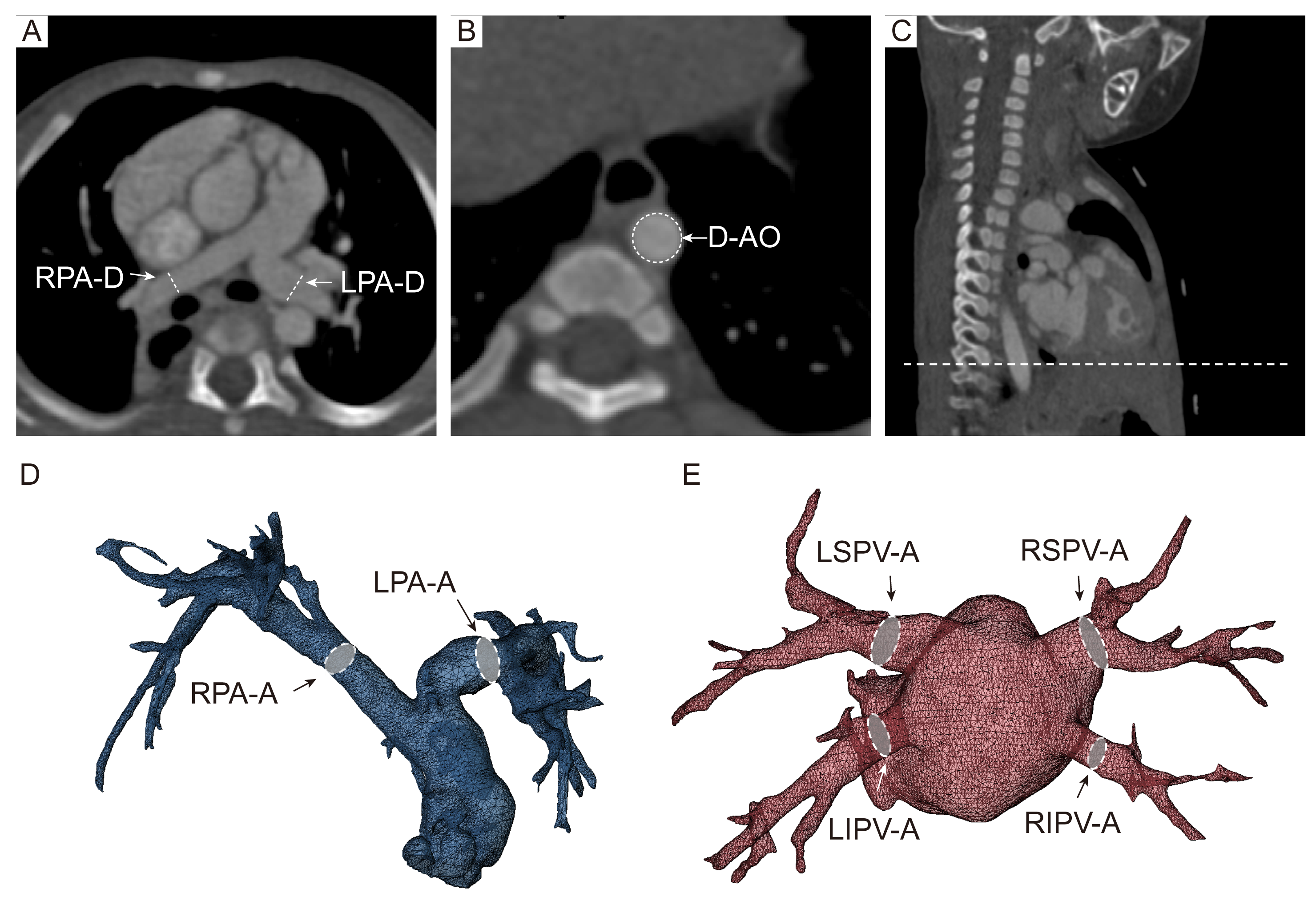

Supplement: Supplementary Figure 1 — Measurement of PA diameter, aorta diameter, PA area, and PV area. (A) Diameters of the left and right pulmonary arteries (LPA-D, RPA-D), measured in the axial plane. (B) Diameter of the descending aorta (D-AO) at the diaphragmatic level in the axial plane. (C) Depicts the measurement position for the descending aorta on the sagittal plane. (D) Areas of the left and right pulmonary arteries (LPA-A, RPA-A) on a 3D reconstructed view. (E) Areas of the left and right superior PV (LSPV-A, RSPV-A) and left and right inferior PV (LIPV-A, RIPV-A) on the 3D reconstructed view. [file Image_1.TIF]

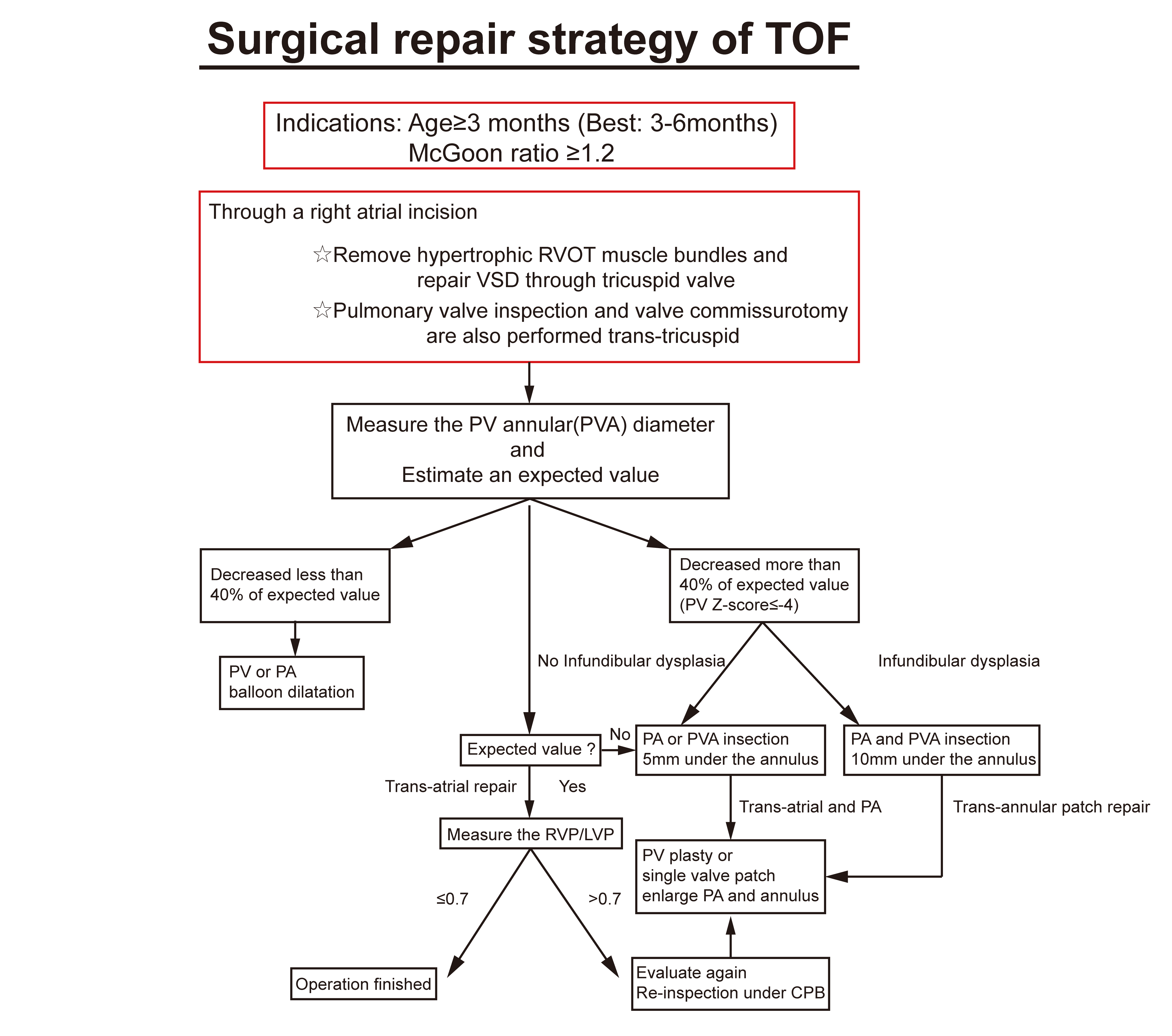

Supplement: Supplementary Figure 2 — Schematic diagram showing our surgical repair strategy for TOF. RVOT, right ventricle outflow tract; VSD, ventricular septal defect; PV, pulmonary valve; PVA, pulmonary valve annular; PA, pulmonary artery; CPB, cardiopulmonary bypass. [file Image_2.tif]

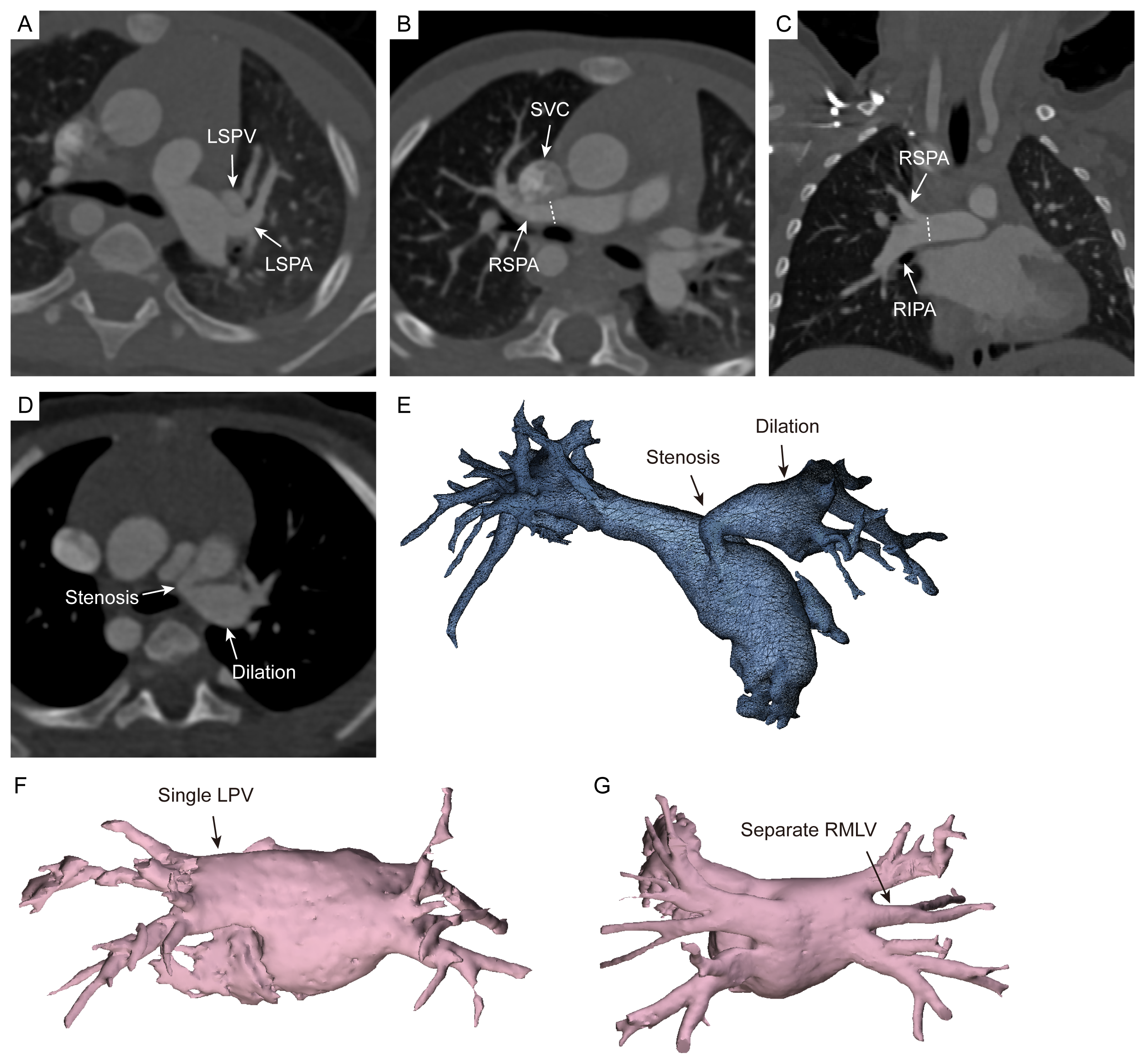

Supplement: Supplementary Figure 3 — The first branch of the left pulmonary artery commonly surrounds the back of the left superior pulmonary vein and moves toward the outside (A). The right superior and inferior pulmonary arteries originate inside the pericardia and moved upward and downward, respectively (B,C) which may be difficult to distinguish in the axial view. (D,E) indicate apparent stenosis and stenotic dilation. (F) indicates a single left pulmonary vein. (G) indicates a separate right middle lobe vein. [file Image_3.tif]
