# Supplementary material for: Pulmonary Vein Index Is Associated With Early Prognosis of Surgical Treatment for Tetralogy of Fallot
Source: Front Pediatr. 2021 Jul 12;9:705553. doi: 10.3389/fped.2021.705553 (PMC8311495; doi:10.3389/fped.2021.705553)
Supplement: Supplementary Table 1 — Reliability analysis of CTA measurements with same methods between two independent radiologists. [file Data_Sheet_1.docx]

| **Supplementary Table 1. Reliability analysis of CTA measurements with same methods between two independent radiologists** | | |
| --- | --- | --- |
| **Measurements** | **ICC ( 95%CI )** | ***P* value** |
| **McGoon ratio** | 0.876 (0.754-0.901 ) | <0.001 |
| **Nakata index** | 0.895 (0.828-0.945) | <0.001 |
| **PVI** | 0.912 (0.608-0.973) | <0.001 |

**Supplementary Table 1.** Reliability analysis results of CTA measurements between the two independent radiologists were reported by ICC (Intraclass correlation coefficients) and 95% CI (Confidence interval). ICC>0.7 was considered valid. CTA, Computed tomography angiography. PVI, Pulmonary vein index.

| **Supplementary Table 2. Univariate analysis of Risk factors for postoperative parameters** | | | | | | |
| --- | --- | --- | --- | --- | --- | --- |
| **Variables** | **PHS** | | **ICU Stay** | | **Ventilator time** | |
|  | **HR (95% CI)** | ***P* value** | **HR(95% CI)** | ***P* value** | **HR(95% CI)** | ***P* value** |
| Age ( y ) | 1.05 (1.01-1.09) | 0.014 | 1.132 (1.085-1.181) | <0.001 | 1.115 (1.069-1.62) | <0.001 |
| Gender ( male vs female) | 0.96 (0.76-1.23) | 0.76 | 1.088 (0.854-1.385) | 0.496 | 0.969 (0.761-1.23) | 0.795 |
| Weight ( kg ) | 1.03 (1.01-1.05) | 0.006 | 1.084 (1.062-1.107) | <0.001 | 1.064 (1.044-1.085) | <0.001 |
| Preoperative SPO_2_ | 1.04 (1.02-1.05) | <0.001 | 1.039 (1.026-1.053) | <0.001 | 1.039 (1.025-1.052) | <0.001 |
| **CTA parameters** |  |  |  |  |  |  |
| McGoon ratio | 1.77 (1.29-2.42) | <0.001 | 1.922 (1.392-2.654) | <0.001 | 1.832 (1.337-2.7510) | <0.001 |
| Nakata index | 1.002 (1.001-1.003) | <0.001 | 1.002 (1.001-1.004) | <0.001 | 1.002 (1.001-1.004) | <0.001 |
| PVI | 1.004 (1.003-1.005) | <0.001 | 1.004 (1.003-1.005) | <0.001 | 1.004 (1.003-1.005) | <0.001 |
| **TTE parameters** |  |  |  |  |  |  |
| RVOT diameter | 1.07 (1.02-1.13) | 0.009 | 1.089 (1.037-1.145) | 0.001 | 1.119 (1.065-1.175) | <0.001 |
| LEDVI | 1.011 (1.006-1.016) | <0.001 | 1.010 (1.005-1.015) | <0.001 | 1.012 (1.008-1.017) | <0.01 |
| **Operative parameters** |  |  |  |  |  |  |
| CPB time | 0.989 (0.985-0.993) | <0.001 | 0.988 (0.984-0.992) | <0.001 | 0.95 (0.981-0.989) | <0.001 |
| Clamping | 0.984 (0.978-0.990) | <0.001 | 0.98 (0.979-0.990) | <0.001 | 0.979 (0.972-0.985) | <0.001 |
| Trans-annular patch, yes or no | 0.568 (0.446-0.723) | <0.001 | 0.586 (0.459-0.747) | <0.001 | 0.601 (0.470-0.768) | <0.001 |
| MAPCAs treatment, yes or no | 0.518 (0.323-0.831) | 0.006 | 0.375 (0.231-0.609) | <0.001 | 0.373 (0.229-0.606) | <0.001 |
| Postoperative RVP/LVP | 0.093 (0.036-0.239) | <0.001 | 0.050 (0.020-0.127) | <0.001 | 0.057 (0.023-0.141) | <0.001 |

**Supplementary Table 2.** Results of univariate analysis, Cox proportional hazards model for PHS, CICU Stay, and Ventilator time were reported by HR (Hazard ratios) and 95% CI (Confidence intervals). Variables with P<0.2 in univariate analysis were selected for Multivariate analysis (yellow background). Age and Nakata index were exclude, as they were collinear with McGoon ratio and body weight, respectively. * indicates statistical significant variables in Multivariate analysis. PHS, Postoperative hospital stay. CICU, Cardiac intensive care unit. SPO2, Percutaneous oxygen saturation. PVI, Pulmonary vein index. LVEDVI, Left ventricular end diastolic volume index. CPB, Cadiaopulmonary bypass. MAPCAs, Major aortapulmonary collateral arteries. RVP/LVP, Postoperative pressure ratio between right and left ventricle.

| **Supplementary Table 3. Linear stepwise regression analysis of risk factors for VIS** | | | | |
| --- | --- | --- | --- | --- |
| **Variables** | **Pearson r** | **Crude OR (95% CI)** | **Adjusted OR** | ***P* value** |
| Age ( y ) | -0.153* |  |  |  |
| Gender ( male vs female) | -0.108 |  |  |  |
| Weight ( kg ) | -0.175** | -0.200 (-0.337, -0.062) | -0.141 | 0.005 |
| Preoperative SPO_2_ | -0.370** | -0.088 (-0.172, -0.003) | -0.120 | 0.043 |
| **CTA parameters** |  |  |  |  |
| Mcgoon ratio | -0.267** |  |  |  |
| Nakata index | -0.237** |  |  |  |
| PVI | -0.418** | -0.015 (-0.022, -0.007) | -0.222 | <0.001 |
| **TTE parameters** |  |  |  |  |
| RVOT diameter | -0.225** |  |  |  |
| LEDVI | -0.290** |  |  |  |
| **Operative parameters** |  |  |  |  |
| CPB time | 0.448** | 0.074 (0.050, 0.098) | 0.319 | <0.001 |
| Clamping | 0.453** |  |  |  |
| Trans-annular patch, yes or no | 0.255** |  |  |  |
| MAPCAs treatment, yes or no | 0.212** | 4.03 (0.93, 7.13) | 0.13 | 0.011 |
| Postoperative RVP/LVP | 0.321** | 8.282 (2.069, 14.495) | 0.138 | 0.009 |

**Supplementary Table 3.** Results of Linear stepwise regression model for VIS were reported by Pearson correlation, Crude OR (Odds ratios), 95% CI (Confidence intervals) and Adjusted OR. Variables that significant correlated with VIS were selected for stepwise regression (yellow background). Age and Nakata index were excluded, as they were collinear with Weight and Mcgoon ratio, respectively. * indicate *P* value < 0.05, ** indicate *P* value < 0.01. SPO_2_, Percutaneous oxygen saturation. PVI, Pulmonary vein index. RVOT, Right ventricular outflow tract. LVEDVI, Left ventricular end diastolic volume index. CPB, Cadiaopulmonary bypass. MAPCAs, Major aortapulmonary collateral arteries. RVP/LVP, Postoperative pressure ratio between right and left ventric.

| **Supplementary Table 4. Univariate logistic regression analysis of Risk factors for major postoperative complications** | | | | | | |
| --- | --- | --- | --- | --- | --- | --- |
| **Variables** | **Serous effusion** | | **Delayed sternal closure** | | **Need for peritoneal dialysis** | |
|  | **Crude OR (95% CI)** | ***P* value** | **Crude OR (95% CI)** | ***P* value** | **Crude OR (95% CI)** | ***P* value** |
| Age ( y ) | 0.946 (0.839-1.067) | 0.368 | 0.360 (0.157-0.825) | 0.016 | 0.454 (0.212-0.974) | 0.043 |
| Gender ( male vs female) | 1.007 (0.553-1.834) | 0.981 | 1.262 (0.482-3.300) | 0.636 | 0.766 (0.255-2.304) | 0.636 |
| Weight ( kg ) | 0.969 (0.908-1.033) | 0.335 | 0.611 (0.463-0.806) | <0.001 | 0.685 (0.524-0.896) | 0.006 |
| Preoperative SPO_2_ | 0.949 (0.923-0.975) | <0.001 | 0.923 (0.885-0.962) | <0.001 | 0.940 (0.901-0.982) | 0.005 |
| **CTA parameters** |  |  |  |  |  |  |
| Mcgoon ratio | 0.377 (0.149-0.949) | 0.038 | 0.165 (0.034-0.799) | 0.025 | 0.255 (0.048-1.353) | 0.109 |
| Nakata index | 0.995 (0.992-0.999) | 0.022 | 0.993 (0.986-1.000) | 0.057 | 0.997 (0.991-1.004) | 0.431 |
| PVI | 0.993 (0.990-0.996) | <0.001 | 0.984 (0.976-0.992) | <0.001 | 0.986 (0.978-0.994) | 0.001 |
| **TTE parameters** |  |  |  |  |  |  |
| RVOT diameter | 0.908 (0.778-1.059) | 0.220 | 0.561 (0.365-0.863) | 0.009 | 0.772 (0.542-1.098) | 0.150 |
| LEDVI | 0.965 (0.945-0.986) | 0.001 | 0.932 (0.888-0.978) | 0.004 | 0.964 (0.927-1.004) | 0.074 |
| **Operative parameters** |  |  |  |  |  |  |
| CPB time | 1.014 (1.006-1.023) | 0.001 | 1.024 (1.014-1.035) | <0.001 | 1.019 (1.009-1.029) | <0.001 |
| Clamping | 1.020 (1.007-1.034) | 0.003 | 1.048 (1.029-1.068) | <0.001 | 1.037 (1.018-1.057) | <0.001 |
| Trans-annular patch, yes or no | 2.019 (1.088-3.749) | 0.026 | 3.106 (0.996-9.679) | 0.051 | 2.390 (0.742-7.692) | 0.144 |
| MAPCAs treatment, yes or no | 1.478 (0.509-4.290) | 0.473 | 4.500 (1.328-15.253) | 0.016 | 3.735 (0.961-14.511) | 0.057 |
| Postoperative RVP/LVP | 30.415 (3.000-308.319) | 0.004 | 2978.805 (69.580-127525.439) | <0.001 | 413.156 (9.310-18334.590) | 0.002 |

**Supplementary Table 4.** Results of univariate logistic regression analysis for serous effusion, delayed sternal closure, and peritoneal dialysis were reported as Crude OR (Odds ratios) and 95% CI (Confidence intervals). Variables with *P*<0.2 in univariate analysis were selected for Multivariate analysis (yellow background). Nakata index was exclude, as it was collinear with McGoon ratio. SPO_2_, Percutaneous oxygen saturation. PVI, Pulmonary vein index. RVOT, Right ventricular outflow tract. LVEDVI, Left ventricular end diastolic volume index. CPB, Cadiaopulmonary bypass. MAPCAs, Major aortapulmonary collateral arteries. RVP/LVP, Postoperative pressure ratio between right and left ventricle.
